# Maximally informative foraging by *Caenorhabditis elegans*

**DOI:** 10.7554/eLife.04220

**Published:** 2014-12-09

**Authors:** Adam J Calhoun, Sreekanth H Chalasani, Tatyana O Sharpee

**Affiliations:** 1 Neurosciences Graduate Program, University of California, San Diego, La Jolla, United States; 2 Molecular Neurobiology Laboratory, Salk Institute for Biological Studies, La Jolla, United States; 3 Computational Neurobiology Laboratory, Salk Institute for Biological Studies, La Jolla, United States; Universidad Nacional Autonoma de Mexico, Mexico

**Keywords:** decision making, information theory, drift-diffusion model, *C. elegans*

## Abstract

Animals have evolved intricate search strategies to find new sources of food. Here, we analyze a complex food seeking behavior in the nematode *Caenorhabditis elegans* (*C. elegans*) to derive a general theory describing different searches. We show that *C. elegans*, like many other animals, uses a multi-stage search for food, where they initially explore a small area intensively (‘local search’) before switching to explore a much larger area (‘global search’). We demonstrate that these search strategies as well as the transition between them can be quantitatively explained by a maximally informative search strategy, where the searcher seeks to continuously maximize information about the target. Although performing maximally informative search is computationally demanding, we show that a drift-diffusion model can approximate it successfully with just three neurons. Our study reveals how the maximally informative search strategy can be implemented and adopted to different search conditions.

**DOI:** http://dx.doi.org/10.7554/eLife.04220.001

## eLife digest

How an animal forages for food can make the difference between life and death, and there are several different searching strategies that may be adopted. Foraging could be more productive if animals could take into account any of the patterns with which food is distributed in their environment, but how much could they measure and memorize? Calhoun et al. show that a tiny worm called *Caenorhabditis elegans* can keep track of how its previous food finds were spread out, and uses this knowledge to optimize future searches for food.

When *C. elegans* forages, it begins by performing an intensive search of where it believes food is likely to be found. This strategy, called ‘local search’, is characterised by the worm making numerous sharp turns that keep it in its target search area. If the worm has not found food after 15 min, it abruptly switches its behavior to a so-called ‘global search’ strategy, which features fewer sharp turns and more forays into the surrounding area.

*C. elegans* is often thought to follow the smell of a food source in order to locate it. While reliable on small scale, this strategy can prove problematic when the distribution of food is patchy. Calhoun et al. show that in extreme conditions, such as when food is completely removed, the animals determine where and for how long to persist with their search based on their knowledge of what was typical of their environment. Such a strategy is called infotaxis, which literally means ‘guided by information’. While the neural circuits underpinning these behaviors remain to be found, Calhoun et al. propose a model that suggests that these circuits could be relatively simple, and made up of as few as three neurons.

**DOI:** http://dx.doi.org/10.7554/eLife.04220.002

## Introduction

In considering animal behavior and decision-making, it is exciting to consider the proposal (Polani, 2009; Tishby and Polani, 2011) that animals may be guided by fundamental statistical quantities, such as the maximization of Shannon mutual information (Cover and Thomas, 1991). The advantage of mutual information as a measure is that its maximization encompasses optimization according to many other statistical measures, such as the peak height or the variance of the distribution. These other measures would give valid results only in certain contexts, such as for predominantly unimodal or Gaussian probability distributions underlying the decision variables. The fact that mutual information can be used with different types of probability distributions makes it possible to quantitatively compare the efficiency of behavioral decisions across species, sensory modalities, and tasks. Indeed, this idea has already yielded insights into diverse behaviors including human eye movements patterns (Najemnik and Geisler, 2005) and animal navigation in a turbulent environment (Vergassola et al., 2007; Masson et al., 2009). Both these patterns of behavior can be accounted for by adapting a maximally informative search strategy to the appropriate behavioral context. This model allocates some actions to improving the estimate of the goal's position rather than directly moving the animal towards the goal (Najemnik and Geisler, 2005; Vergassola et al., 2007). In these contexts, behavioral analyses have shown that strategies aimed at moving directly toward a goal are unable to explain key features of the animal's response. For example, humans sometimes make saccades to examine a region between, rather than directly at, the two likely locations for a target (Najemnik and Geisler, 2005). Similarly, birds and moths zigzag perpendicular to the wind direction to find the source of an odor plume (Vergassola et al., 2007). Information-maximization (‘infotaxis’) is consistent with direct strategies such as chemotaxis in certain conditions. When the information content of the environment is very high, such as when chemical gradients can be tracked reliably, strategies based on information maximization converge to chemotaxis (Vergassola et al., 2007). Thus, an information maximization approach can be viewed as a generalization of following direct sensory gradients to a broader and more challenging set of behavioral tasks.

**Figure 1.**
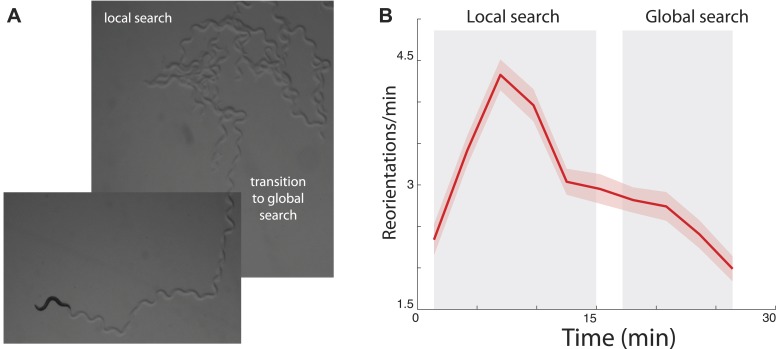
Transition between local and global search in *C. elegans* foraging trajectories following their removal from food. (**A**) Animals search the local area by producing a large number of turns before abruptly transitioning to a global search. (**B**) Across many animals, this transition is readily apparent in the mean turning rate. Standard error of the mean is shown as the lightly shaded region around the solid average line. **DOI:** http://dx.doi.org/10.7554/eLife.04220.003

Among the multitude of decisions that animals make throughout the day, foraging for food is perhaps the most challenging and critical for survival. Interestingly, a number of species have been reported to spend more time in areas where they have recently observed food (Karieva and Odell, 1987; Benedix, 1993), suggesting that there might be an underlying logic to search that generalizes across species. Recent experimental studies have observed similar foraging patterns in the nematode *Caenorhabditis elegans* (Hills et al., 2004; Wakabayashi et al., 2004; Gray et al., 2005; Chalasani et al., 2007). After removal from food, the animal first performs an intense search around the area where it believes food is likely to be located (Figure 1A). This period is characterized by an increased number of abrupt turns allowing the animal to stay in the proximal area (Figure 1B) and is termed ‘local search’. After approximately 15 min, animals reduce their number of turns to a basal rate (Figure 1B). This produces more extended trajectories (Figure 1A) and allows the animal to leave the proximal zone and explore a much larger area (‘global search’). Although *C. elegans* is traditionally considered to be a chemotactic searcher (Ferree and Lockery, 1999; Pierce-Shimomura et al., 1999; Iino and Yoshida, 2009), moving up or down chemical gradients to find the source of an odorant, in these conditions animals have no chemical gradient to follow. We set out to explore whether a single underlying strategy could explain the different aspects of food search behavior in this well-studied model.

## Results

### Maximally informative search strategies describe both local and global search states

**Figure 2.**
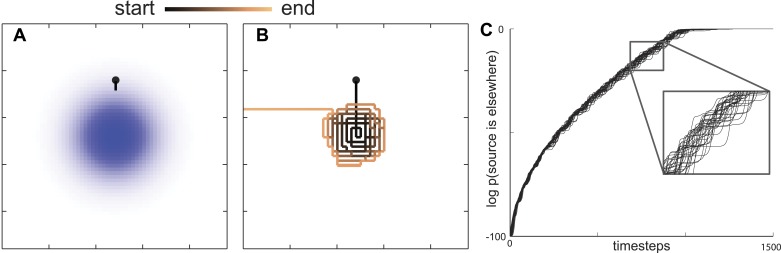
Maximally informative trajectories exhibit abrupt transitions between spiral-like and straight motion towards the boundary. (**A**) Initial trajectories of the model head directly towards the peak probability of finding an odor source. (**B**) After some period of time the model displays an abrupt transition in behavior from a spiral-like motion to a straight motion towards the boundary. (**C**) The log probability that the food is elsewhere consistently increases as the search progresses. The transition between local (spiral-like) and global (straight motion towards the boundary) search occurs when this probability approaches 1. **DOI:** http://dx.doi.org/10.7554/eLife.04220.004

Since *C. elegans* performs a food search even in the absence of a gradient (Hills et al., 2004; Wakabayashi et al., 2004; Gray et al., 2005), they must have a prior belief about how food is distributed in the environment. For the sake of simplicity, we assume that the probability of finding food is initially distributed as a two-dimensional Gaussian distribution, which imposes the minimal structural constraint beyond the variance of the spatial distribution (Jaynes, 2003). When searching through this space, an animal using the maximally informative trajectory should move in the direction that maximizes its information about the location of food. This can be calculated by taking into account the probability that the nearby environment would emit food odor and then estimating the change in information the animal expects will result from any detection or non-detection events (Vergassola et al., 2007). This means that even a non-detection of a food odorant is informative as it lessens the likelihood that food is nearby. Ultimately, the probability of detecting an odorant depends on the likelihood of food sources across the environment $\left(\vec{r}\right)$. Information maximization can be analyzed with respect to this quantity, see ‘Materials and methods’ for details. Analyzing these solutions, we find that the maximally informative trajectories first take the searcher towards the peak in the maximum likelihood of food distribution (Figure 2A) and then follow an outward motion (Figure 2B). This intensive search of a small area is qualitatively consistent with the local search performed by *C. elegans*.

Given an infinitely sized arena, this spiral-like motion would continue indefinitely (Barbieri et al., 2011). However, searchers only have knowledge of a finite area (the full extent of the area shown in Figure 2A–B). Further, it is not necessarily true that there will always be food near where it has been seen before. Thus, we have to allow for the possibility that food will *not* be in the nearby area. In mathematical terms, we allow for the probability that a food source is in the nearby area to deviate from 1. Initially, this probability was set to be very close to one (within numerical accuracy, deviation from 1 was ∼10^−100^). However as the search progressed, and no odorants were detected, this probability decreased according to the Bayesian rule:

(1) $$p_{t+1}\left(A|n=0\right)=p_{t}\left(A\right)\frac{P(n=0|A)}{P(n=0)},$$

where $p_{t+1}\left(A\right)=p_{t+1}\left(A|n=0\right)$ is the updated probability given that *n = 0* odor detections were observed. The update rule in Equation (1) reflects the fact that, while the searcher at each step expects to detect a certain number of odorants, none are detected because the source is absent. While initially $p_{0}\left(A\right)$ is set very close to 1, it gradually decreased to zero. We found that allowing the probability to decrease during the search causes the local search to consistently end abruptly at locations that were very far from the boundaries of modeled area A (Figure 2B). The abrupt transition occurred for any initial values of $p_{0}\left(A\right)$ as long as it was not identically equal to 1 at the start of the search. [If $p_{0}\left(A\right)=1$, then the probability to find food outside of the local area is zero and it will remain so even after the Bayesian update in Equation (1)]. After the transition, the search trajectory would then follow a straight path to the boundary of the modeled area (Figure 2A–B). These features of search trajectories are consistent with *C. elegans* transitioning between local and global search.

### Maximally informative strategies quantitatively describe foraging trajectories

One interesting feature of this maximally informative search strategy is the abruptness of the transition. Movement around the peak initial belief is followed by a sudden switch to motion away from it. The transition from local to global search corresponds, at least in the model, with the searcher's estimate that the probability that food is located elsewhere equals 1 (Figure 2C). This indicates that qualitative changes in behavioral state arise due to beliefs that no longer reflect old information.

**Figure 3.**
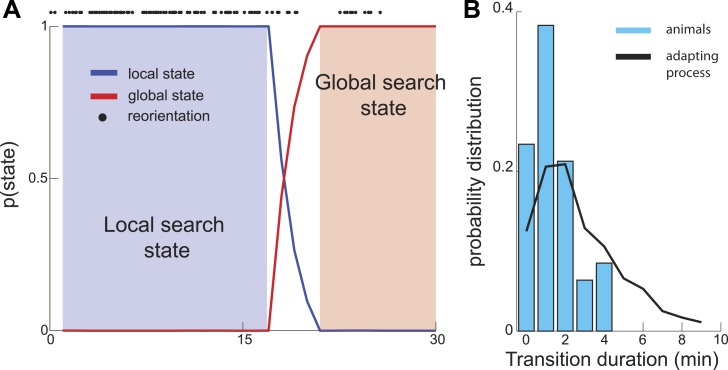
Sharp Transition between local and global phases of the search. We used a Hidden Markov model to estimate the probability that animal's behavior falls into one of two states. (**A**) Example analysis based on a single trajectory shows fast (several minutes) switching time between the local and global phases of the search. (**B**) The distribution of transition durations across a set of trajectories from different animals. **DOI:** http://dx.doi.org/10.7554/eLife.04220.005

As described above, in the maximally informative model, the transition between the local and global states of the search occurs abruptly. In experiments, the reduction in the number of turns appears to occur gradually (Figure 1B). However this difference could be an artifact of the averaging of trajectories across multiple worms. In contrast, individual worm trajectories could still have sharply defined transitions, as can be seen in Figure 1A. To investigate the sharpness of this transition within individual worm trajectories, we applied a Hidden Markov model framework (Abeles et al., 1995; Seidemann et al., 1996; Bishop, 2004; Jones et al., 2007; Miller and Katz, 2010) to experimentally recorded trajectories. If segments of single-animal trajectories represent mixtures of states corresponding to local and global parts of the search, then the probability of observing global search patterns will increase gradually. However, analysis of experimental traces revealed a sharp transition between local and global search states on the order of a few minutes (Figure 3A,B). Thus, the search trajectories both in experiment and theory exhibit a sharp transition between the local and global parts of the search.

**Figure 4.**
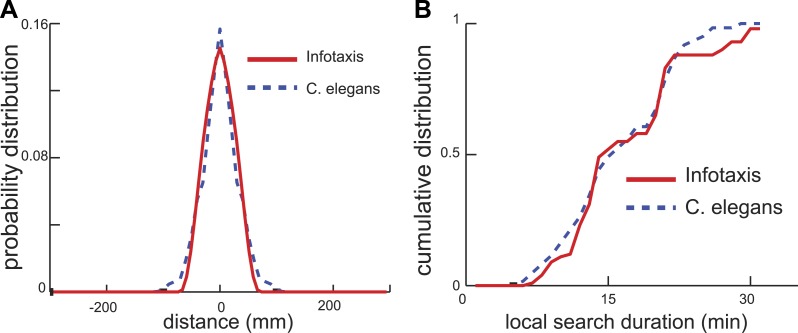
Infotaxis model quantitatively accounts for the worm trajectories. (**A**) The distribution of worm displacements from an initial position at the end of the local search is non-Gaussian and can be fitted using the three parameters (See ‘Materials and methods’) of the infotaxis model. (**B**) The same set of parameters also accounts for the cumulative distribution for the local search duration across different individual worms. **DOI:** http://dx.doi.org/10.7554/eLife.04220.006

Next, we examined whether the infotaxis framework could quantitatively account for the distribution of worm search trajectories. The infotaxis model contains three independent parameters: the width of the initial prior probability distribution, filter length representing physical parameters, and how close the initial values for $p_{0}\left(A\right)$ was set to 1 (See ‘Materials and methods’). Fitting these parameters of the infotaxis model, it is possible to quantitatively account for the experimental distribution of worm positions at the end of local search (Figure 4A). Importantly, the same set values of these parameters adjusted to match the spatial distribution (Figure 4A) also produced (without re-adjustment) the cumulative distribution of local search duration and matched experimental measurements (Figure 4B, two-sample Kolmogorov–Smirnov test, p = 0.45). The conversion between the spatial axis in Figure 4A and the temporal scale in Figure 4B is set by the calculated value of the worm's speed (∼0.17 mm/s), and does not represent an adjustable parameter. Thus, the infotaxis model can quantitatively account for the properties of worm search behavior after removal from food.

**Figure 4—figure supplement 1.**
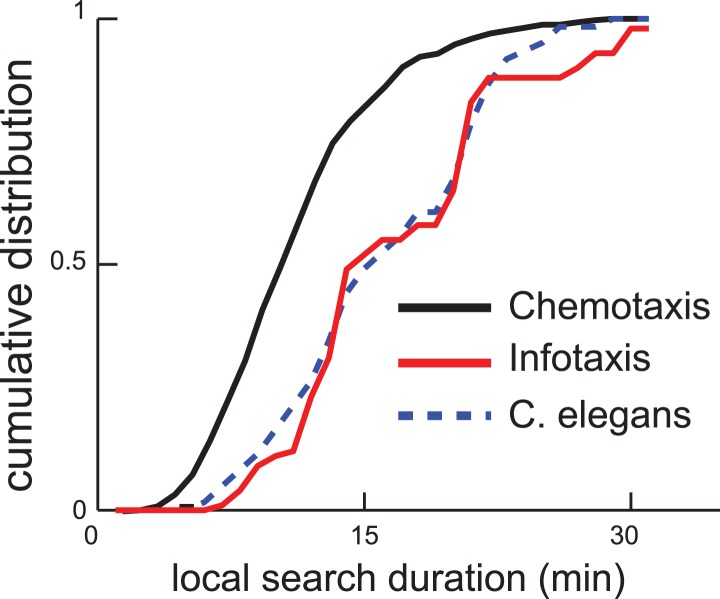
Comparison of measured local search duration times with predictions based on chemotaxis and infotaxis models. The chemotaxis model was fit by measuring the maximum and steady-state turning rates of the animal and constraining the mean turning rate. The data (blue dashed line) and infotaxis (red solid line) are reproduced from Figure 4. **DOI:** http://dx.doi.org/10.7554/eLife.04220.007

### Comparison with chemotaxis model

**Figure 5.**
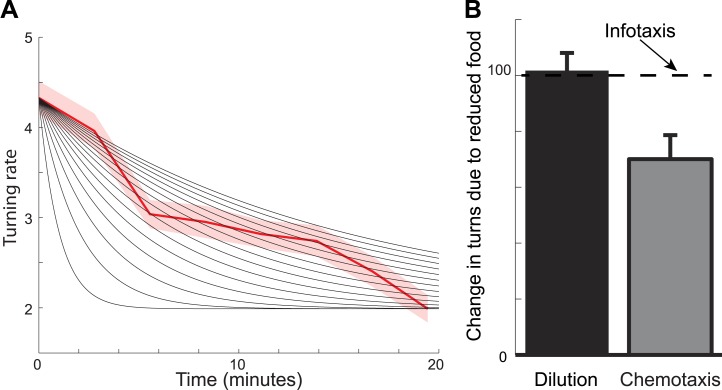
Foraging trajectories deviate from predictions of the chemotaxis model. The chemotaxis model explains the reduction in the average number of turns as adaptation to low odorant concentration. (**A**) For different adaptation times, the predicted dynamics of turn rate can match either the slow decay in the beginning of the search or the small rate of turning at the end of the search, but not both. Black lines are predictions using adaptation while red shows experimental measurements. (**B**) The average number of turns is unaffected by changes in food concentration (black), in contrast to chemotaxis predictions (grey bar) and in agreement with the infotaxis predictions (dashed line). **DOI:** http://dx.doi.org/10.7554/eLife.04220.008

One may wonder whether other search strategies could also account for food search behavior in worms. Among these, chemotaxis represents the most widely used and parsimonious model of animal behavior (Brown and Berg, 1974; Pierce-Shimomura et al., 1999, 2005; Iino and Yoshida, 2009). A searcher using this strategy would be expected to transiently increase its turning rate when removed from food due to a sudden, large change in food gradient. The subsequent decline in the number of turns would then be explained by adaptation to the low (zero) odorant concentration. Although this explanation seems plausible, it could not quantitatively account for three properties of foraging trajectories: (i) the long duration of the local search, (ii) the rapid exit from the local search state, and (iii) the inability of food concentration to influence local search. An explanation based on adaptation with a single time constant could be ruled out based on the juxtaposition between the relatively long duration of the local search with the fairly rapid transition between the local and global phases of the search. Adaptation with a slow time constant could explain the fairly substantial duration of the local search but not its sharp transition to a global search. On the other hand, adaptation with a short time constant could match the low number of turns during the global search but would underestimate the number of turns and the duration of the local phase of the search (Figure 5A). Quantitatively, adjusting the adaptation time constant to match the observed durations of the local search phase produces trajectories with much broader transitions between local and global phases of the search than is observed experimentally (Figure 3B, comparison between the solid line and histograms, see also Figure 4—figure supplement 1).

Perhaps a more striking illustration as to why chemotaxis does not fully describe the foraging trajectories comes from experiments where worms are transferred from patches of food of the same size but with different concentrations. The chemotaxis model makes predictions based on the change in odorant concentration. This change will be smaller for animals that are removed from patches with more diluted food. Therefore, the chemotaxis model in this case would predict that animals will make a smaller number of turns (Figure 5B). In contrast, the infotaxis model makes predictions based not on the last odorant concentration that the animal experienced prior to its removal from food, but on the relative distribution of food in the environment. The spatial variance of this distribution is not affected by the dilution. Therefore, the infotaxis model would predict that the animals will make the same number of turns regardless of the bacteria concentration within the lawn, provided the lawns have the same size. This prediction was supported by our measurements (Figure 5B). Overall, we have found that *C. elegans* behavior when removed from food cannot be explained as chemotaxis but is consistent with infotaxis.

### Drift-diffusion approximation to the maximally informative search

The results we have presented so far argue that the quantitative characteristics of animals' behavior match what would be expected for an optimal, maximally informative (Vergassola et al., 2007) or (equivalently) Bayesian (Najemnik and Geisler, 2005) model of search. This model continuously updates the likelihood of a food source being present throughout the duration of search. At first glance, these calculations require the ability to maintain and update the corresponding “mental maps” of the environment. However, the animals could also approximate complex computations with empirically-tuned simple search heuristics that have only slightly smaller than maximal yields. Interestingly, as the search progresses, the log probability $1-p_{t}\left(A\right)$ that the food is located elsewhere accumulates. Broader priors require more time to come to the conclusion that food is located elsewhere. When this probability reaches one, the local phase of the search ends and the global phase begins. The approximately linear increase in the log probability $1-p_{t}\left(A\right)$ observed during most of the local search duration (after the initial period of supra-linear increase, cf. Figure 2C) suggests that the timing of the transition from the local to global search could be accounted for by a simple drift-diffusion model (Bogacs et al., 2006; Insabato et al., 2006). In our set-up the drift-diffusion model has effectively only one parameter—the drift rate. While in most applications drift-diffusion models are considered together with an adjustable threshold, here the lower threshold value is fixed to 0 because the dynamical variable represents probability. Similarly, the starting value of this probability (which we set to be just under 1) also has relatively weak influence on the duration of local search. This is because the initial decrease in $\text{ln}[1-p_{t}\left(A\right)]$ occurs supra-linearly before settling on the linear increase.

**Figure 6.**
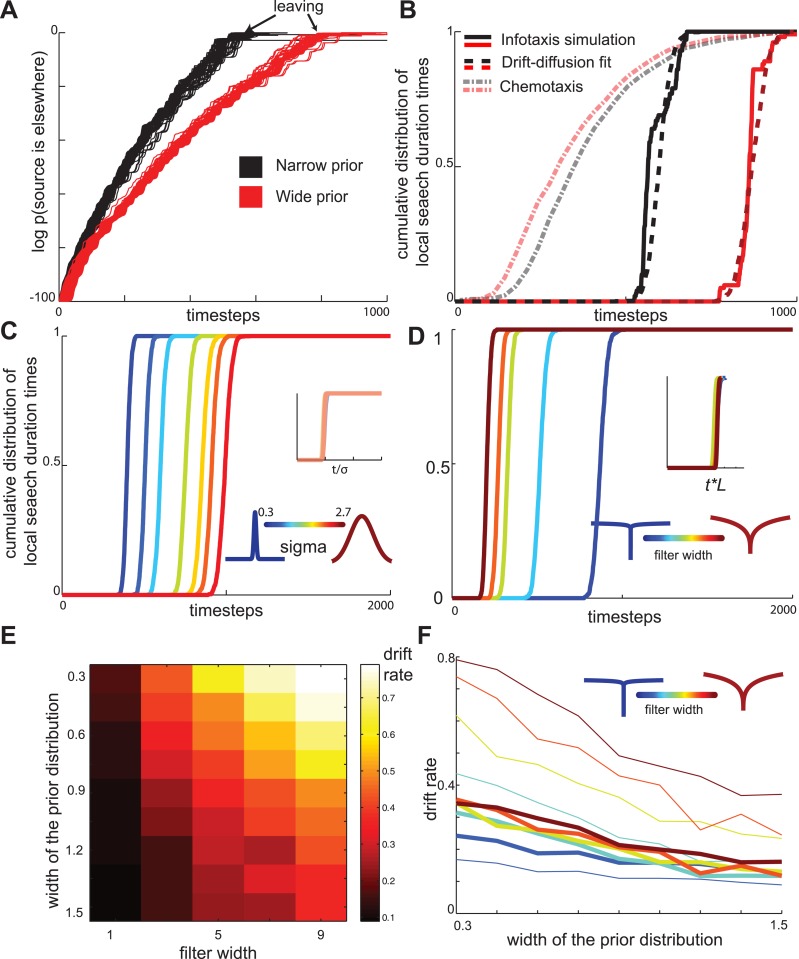
Drift-diffusion approximates maximally informative search across a range of conditions. (**A**) The log probability that the food is located elsewhere has approximately linear dynamics, resembling a drift-diffusion decision variable. The drift rate (slope) decreases with the width of the prior distribution (**B**) The distribution of local search duration times can be approximated by a drift diffusion model for a range of conditions. The chemotaxis model predicts an opposite shift in the local search duration times between wide and narrow priors compared to the infotaxis predictions. In both panels (**A**) and (**B**) red and black curves correspond to wide and narrow priors, respectively. (**C**) The drift rate increases linearly with the width σ of the prior distribution. (**D**) The drift rate decreases linearly with filter width *L* (**E**) The drift rate depends primarily on the ratio L/ σ. (**F**) Normalizing models with different filters by their prior distribution widths reveals a common strategy. **DOI:** http://dx.doi.org/10.7554/eLife.04220.009

The key property of the maximally informative foraging strategies is that they depend on the width of the initial (‘prior’) distribution of food in the environment. We find that changing the width of the distribution σ changes the rate at which the evidence that food is elsewhere accumulates (Figure 6A). Adjusting the slope of the drift-diffusion model captures both the change in evidence-accumulation as well as the observed distribution of transition times from local to global search (Figure 6B). Furthermore, the slope of the best-fitting drift-diffusion model scaled linearly with σ (Figure 6C). These observations suggest that animals could empirically learn the appropriate slope for different distributions of food, and in this way perform nearly optimal foraging strategies with minimum computational effort. Notably, the distribution of local search duration times produced by the chemotaxis model show the opposite dependence on the width of the prior distribution compared to the infotaxis model (Figure 6B).

The maximally informative foraging trajectories are affected not only by the width of the prior distribution but also by odorant characteristics. For example, the diffusivity of odorant molecules affects the calculation of the likelihood of food source. Perhaps fortuitously for animals with small neural circuits, we found that the changes in diffusivity primarily affected the rate of increase $\text{ln}[1-p_{t}\left(A\right)]$, but the overall dynamics could still be described by the that drift-diffusion model (Figure 6D). The slope of the drift-diffusion model increased approximately linearly (Figure 6D) with the spatial extent *L* of the diffusion filter (Vergassola et al., 2007), see also Equation 3 in ‘Materials and methods’. Notably, the drift rate depends primarily on the ratio σ/*L* (Figure 6E–F). These results demonstrate that maximally informative foraging trajectories can be approximated by a simple drift-diffusion model across a range of behaviorally relevant conditions.

## Discussion

In this work we have shown that the exploratory behavior of a small animal, the nematode *C. elegans*, meets the quantitative benchmarks expected for an optimal, maximally informative strategy. This analysis formally requires continuous updates to the likelihood of food sources based on incoming sensory inputs. At the same time, we find that the resulting maximally informative foraging strategies can also be implemented with a combination of a random confined walk with an internal drift-diffusion variable to encode the transition to a new search strategy. The presence of such transitions illustrates how emergent discrete decisions occur as a result of continuous exploration of the environment. Previous studies have shown that drift-diffusion models can provide a substrate for optimal calculations in two alternative forced-choice tasks (Bogacs et al., 2006). Here, we find that these models can also help approximate optimal calculations in cases where alternative choices are not imposed externally but emerge as an intrinsic part of behaviors that optimize gain over long time scales.

### A tentative circuit

**Figure 7.**
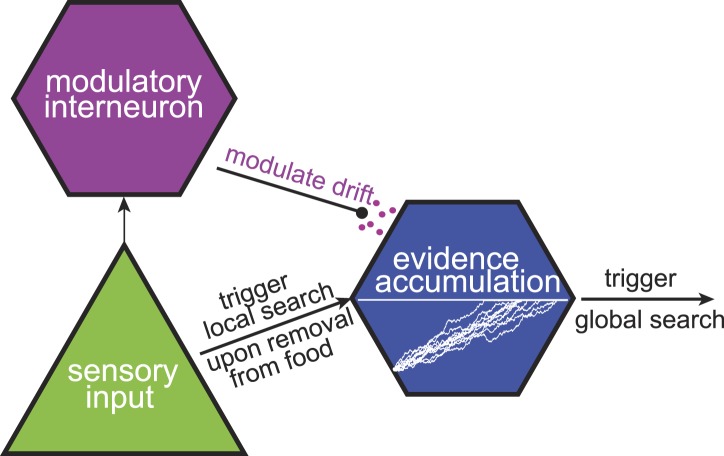
A tentative neural model for near-optimal foraging. Maximally informative foraging can be approximated by a combination of local and global search phases. Responses of a sensory neuron initiate the start of the local search. The passage of time during the local search is encoded in the intracellular voltage of an interneuron. Finally, the duration of the local search can be modulated by the release of neuromodulators. **DOI:** http://dx.doi.org/10.7554/eLife.04220.010

The *C. elegans* neuroanatomy (Gray et al., 2005) suggests that multi-phase foraging strategies can be implemented at the neuronal level, even in simple nervous systems. Taking the two-phase foraging circuit (Gray et al., 2005) that we analyzed here as an example, one may hypothesize that the initial trigger for the start of the search is provided by sensory input, likely through the AWC sensory neuron (Figure 7). AWC neurons respond to a decrease in odorant concentration (Chalasani et al., 2007). However, these responses are transient (Chalasani et al., 2007) and do not last long enough to account for the long duration of local search (∼15 min). Instead, we hypothesize that local search is maintained based on the responses of one of the interneurons. The gradual change in state of these neurons that receive sensory input, for example the AIB and AIZ interneurons, may encode the passage of time since the start of local search. Modulating the rate at which the internal state of a neuron changes allows the animal to adjust the duration of local search after sensing aspects of the environment such as how food is spatially distributed and how far the odorant molecules diffuse from this particular food source. We hypothesize that this modulation occurs through neuromodulatory signaling, which is known to be involved in local search behavior (Hills et al., 2004). In summary, a neural circuit based on just a few neurons suffices to implement foraging strategies that approximate the maximally informative, but computationally intensive, decisions.

### Infotaxis vs chemotaxis

There are a wide range of possible foraging strategies that animals might follow. These include chemotaxis based on local sensory cues, different types of random walks (Bartumeus et al., 2002; Humphries et al., 2010; Viswanathan et al., 2011; Humphries et al., 2012), and computationally intensive models based on detailed memories of past experiences. This raises the question of whether the optimal foraging strategy is constrained not only by the physical environment but also by the computational complexity of its implementation (Tishby and Polani, 2011). One approach to this solution is provided by the conventional chemotaxis model (Ferree and Lockery, 1999; Pierce-Shimomura et al., 1999). A chemotactic behavior can be implemented based on responses of just one sensory neuron. However, the resulting search strategies are driven directly by changes in the gradient and do not necessarily reflect the typical size of food patches. While infotaxis and chemotaxis strategies converge under conditions of smoothly varying gradients, this is not so in cases where transitions between patches are common. In fact, we found that chemotactic trajectories exhibited not only a much weaker dependence on the patch size compared to infotaxis trajectories but also predicted the opposite relationship between patch size and search duration (Figure 6B). In addition, we found that worm and foraging trajectories were unaffected by the overall food concentration within the patch (Figure 5B), in agreement with infotaxis but in contrast to chemotaxis predictions. The addition of interneurons to the circuit, as schematized in Figure 7, makes it possible to dissociate the change in the gradient from the duration of local search. Thus, the modest increase in computational cost associated with the addition of interneurons allows for more flexible behavior than would be seen in a simple chemotaxis strategy.

It has been noted that foraging strategies that maximize the mutual information about target locations do not always produce the maximal yield. Such situations have been observed in cases where the targets are mobile (Agarwala et al., 2012). In this case, although the searcher knows precisely where the food is located at a given time, it might not be able to get to the food source before it moves again. Animals might counteract this problem with predictive coding, using foraging strategies that maximize information about the food source location at a sufficient time in the future (Polani, 2009; Tishby and Polani, 2011; Bialek, 2013). So long as the parameters of simpler models can be easily learned through experience, there are no barriers to implementing such strategies with a few neurons. Indeed, including predictive information may take the form of an increased rate of evidence accumulation in an infotaxis-like model.

The fact that both infotaxis and drift-diffusion models can account for the properties of foraging trajectories does not take away from the stated goal that animal behavior is guided by information maximization. After all, the drift-diffusion models are fitted to parameters of infotaxis trajectories. These fits dictate how the animals should adjust search times depending on the typical widths of food patches in the environment. While the infotaxis model predicts that local search should last longer for wider food patches, the chemotaxis model makes the opposite prediction (Figure 6). At the same time, even to set parameters of drift-diffusion models, one would need to estimate the variance of the food distribution across space. This is quite a feat for such a small animal as *C. elegans.* Further, it might be possible that *C. elegans* are capable of adjusting their behavior based on higher-than-second moments of the probability distribution. Demonstrating this would require more fine-scale experiments to control differences in both size and shape of food patches from which the animals are removed. If such sensitivities are observed, they would implicate the involvement of more complicated circuits that could be mapped onto a single drift-diffusion model. Finally, it is worth noting that the local search of *C. elegans* exhibits striking similarities to other invertebrates, such as crabs (Zeil, 1998), bees (Gould, 1986; Dyer, 1991), and ants (Wehner et al., 2002). In particular, the search patterns of desert ants that have been displaced on their return to the nest (Wehner et al., 2002). When arriving near the presumed location of the nest, animals follow a spiral search pattern that is consistent with infotaxis trajectories (Barbieri et al., 2011). Following large displacements, ants have great difficulties finding the nest with local search patterns and transition to a strategy that is reminiscent of the global search executed by *C. elegans* (Wehner, 2003; Wehner et al., 2006). The large-scale foraging patterns in ants are difficult to study quantitatively because of the large areas involved and a few published foraging trajectories (Wehner et al., 2002). Our results add to these by showing that invertebrates can integrate more abstract quantities than spatial position and operate directly on the probability that the food (or nest) is located elsewhere. Importantly, the animals do not need to perform information-theoretic calculations all of the time; instead they can set parameters of the approximating models through learning and experience.

In summary, animals appear to guide their foraging behavior by searching for information. This simple behavioral rule is able to account for multiple search strategies, as well as the emergent transitions between them. While seemingly complex, this strategy can be easily implemented in a reduced neural system. We anticipate that this principle will prove useful as a general theory of search and decision-making in a wide range of contexts.

## Materials and methods

### Quantification of animal behavior

*C. elegans* in the L4 larval stage were allowed to grow overnight on an agar plate containing a 100 μl circular patch of the *E. coli* OP50 strain (OD_600_ = 0.4). For testing, animals were moved to an agar observation plate without any food where they were corralled into a 1″ square by a filter paper soaked in 200 mM CuSO4, which animals generally avoid. Moving an animal requires them to picked up using a metal object. These animals spend roughly 2 min moving forward before initiating their search. Worm movement was recorded for 30 min at three frames per second, and the first 2 min are ignored.

### Computation of infotaxis trajectories

Infotaxis trajectories were modeled using a 128 × 128 grid representing position and probability distribution of the food source in the environment. At each step, the anticipated change in entropy was computed taking into account two possibilities: observing or not observing odorant hits. Although the initial descriptions of the model separated odorant detection events according to the number of odorant hits, in our setup (absent food source) the computation of those probabilities was numerically unstable. This is the reason why we reduce the coding to binary, either ‘no hits’ or ‘a non-zero number of hits’. As such, each change in entropy is calculated using the probability to receive a hit or the probability to receive zero hits. Computations are halted upon being within one space of the border or after no movement for 15 time steps. Trajectories are computed as in (Vergassola et al., 2007). The probability of an odor source being located at location **r**_**0**_ after observing a trace of odor encounters is given by

(2) $$P_{t}\left(r_{0}\right)=\frac{L_{r_{0}}(T_{t})}{\int L_{x}(T_{t})dx}$$

or, alternately,

$$P_{t}\left(r_{0}\right)=\frac{\text{exp}[-\int_{0}^{t}R\left(r\left(t^{'}\right)|r_{0}\right)dt']\overset{H}{\underset{i=1}{\prod }}R(r(t_{i})|r_{0})}{\int \text{exp}[-\int_{0}^{t}R\left(r\left(t^{'}\right)|x\right)dt']\overset{H}{\underset{i=1}{\prod }}R(r(t_{i})|x)dx}$$

where H is the number of hits observed during the trajectory at time *t*_*i*_. Here, $L_{r_{0}}(T_{t})$ is the likelihood of observing the trace *T*_*t*_ odor encounters from a source located at **r**_0_. When a region is visited and the source is not found, that region has its probability set to 0. R is the function representing the mean hit rate observed at location **r** if the source is at **r**_0_. It has the following form:

(3) $$R\left(r|r_{0}\right)=\frac{\rho }{\text{ln}\left(\frac{\sqrt{D\tau }}{a}\right)}K_{0}\left(\frac{\left|r-r_{0}\right|}{\sqrt{D\tau }}\right)$$

where *a* is the size of the searcher, ρ is the particle emission rate, *D* is the diffusivity of the particles, the particles have a finite lifetime τ, and *K*_*0*_ is the modified Bessel function of order 0. The filter width L is defined as $\text{L}=\sqrt{D\tau }$.

During movement, the expected change in entropy when moving is

$$\Delta S\left(r\to r_{j}\right)=P_{t}\left(r_{j}\right)\left[-S\right]+\left[1-P_{t}\left(r_{j}\right)\right]\left[\rho_{0}\left(r_{j}\right)\Delta S_{0}+\rho_{1}\left(r_{j}\right)\Delta S_{1}\right]$$

where $\rho_{0}$ represents the probability that 0 detections are made at ***r***_*j*_ during a timestep and $\rho_{1}$ represents the probability that *any* detections are made. In this cases, the expected number of hits $h\left(r_{j}\right)=\int P_{t}\left(r_{j}\right)R(r_{j}|r_{0})dr_{0}$ with the probability of hits following a Poisson law. In other words, $\rho_{1}=\left(1-p_{t}\left(A\right)\right)(1-e^{-h})$.

During search, while a hit results in a change in the probability landscape of $R\left(r\text{|}r_{0}\right)$, no hits will update the prior by convolving it with $\exp(-R\left(r\text{|}r_{0}\right))$. The length scale of this filter is calculated by fitting it with an exponential function exp(−x/L) with an adjustable length scale *L*.

### Drift-diffusion model

The decision variable was modeled as an accumulating value with initial value set at −100 to represent the log-likelihood that food is elsewhere. Drift and diffusion parameters were extracted from the time series of infotaxis trajectories and decisions were simulated using the following equation:

(4) dx=Adt+cdW.

Here, *x* is the current evidence in favor of a decision. It grows with mean drift rate *A* and Gaussian noise *dW* is drawn with standard deviation *s*. Simulations were ended once the decision variable reached 0.

## Additional files

### Major dataset

The following dataset was generated:

Calhoun AJ, Chalasani SH, Sharpee TO, 2014, Data from: Maximally informative foraging by *Caenorhabditis elegans*, 10.5061/dryad.3j2j9, Available at Dryad Digital Repository under a CC0 Public Domain Dedication.
