# Supplementary material for: Maximally informative foraging by Caenorhabditis elegans
Source: eLife. 2014 Dec 9;3:e04220. doi: 10.7554/eLife.04220 (PMC4358340; doi:10.7554/eLife.04220)
Supplement: Related texts

# Article commentary: Decision letter

**DOI:** 10.7554/eLife.04220.011

**Affiliations:** Universidad Nacional Autonoma de Mexico, Mexico

eLife posts the editorial decision letter and author response on a selection of the published articles (subject to the approval of the authors). An edited version of the letter sent to the authors after peer review is shown, indicating the substantive concerns or comments; minor concerns are not usually shown. Reviewers have the opportunity to discuss the decision before the letter is sent (see review process). Similarly, the author response typically shows only responses to the major concerns raised by the reviewers.

Thank you for sending your work entitled “Maximally informative foraging by *Caenorhabditis elegans*” for consideration at *eLife*. Your article has been favorably evaluated by a Senior editor, a Reviewing editor, and 2 reviewers.

The Reviewing editor and the other reviewers discussed their comments before we reached this decision, and the Reviewing editor has assembled the following comments to help you prepare a revised submission.

The authors studied the foraging behavior of the *C. elegans* worm and found that the transitions from a local (search intensive) to a global (less turns, larger area) search strategy can be explained by a drift diffusion model that effectively maximizes information about the location of the food source. This infotaxis model has greater explanative value as compared to a chemotaxis mechanism that only follows the gradient of odorant concentration and the authors demonstrate that is could be implemented by a three-neuron circuit.

1) However, there are some few issues that the reviewers did not fully understand, and I agree: is the drift-diffusion model described by Equation (3) connected to the worm's sensory data in some way? In particular, decisions are reached when x reaches a value of zero, but is this initial value of x at the start of the local search? One of the reviewers thinks this is not clearly stated, and that might not be true, and I agree.

2) One of the reviewers thinks that the qualitative features (e.g., the abrupt change from local to global search; food concentration independence of optimal infotaxic search) show that this model is distinct from chemotaxis, and that it would be helpful to make direct comparisons with best fits from situations of chemotaxis in the figures (Figures 4a,b, 5B and 6b).

Minor comments/questions:

1) How similar are the foraging behaviors in other invertebrate species mentioned, and are there any other instances of infotaxis/mutual information searching in animal behaviors other than visual saccades and bacteria?

2) It would be interesting if the authors could describe an intuitively appealing rationale for why infotaxis as a method should be predicted to work. For example, when considering foraging behavior and various possible mathematical descriptions, is there a good a priori reason to expect that mutual information search would be favored?

3) The model contains three independent parameters that are fitted to reproduce the experimental distribution of the animal's positions at the end of the local search. Then the authors write, “Importantly, the same set of parameters can also account for the cumulative distribution of the durations of local search (Figure 4b).” Do they mean that the same set of parameters can be used to do this or that the same numerical values of the parameters account for the optimal durations?

4) “Predictions of the infotaxis model are based on the relative distribution of food across space, rather than on its absolute concentration.” Sounds a bit confusing; chemotaxis is based on a gradient, not an absolute concentration.

5) “At first glance, these calculations require the ability to maintain and update such “mental maps”, which may seem too computationally demanding to be implemented in a small neural circuit, such as the one found in *C. elegans*. Therefore, one may wonder whether the animals approximate the maximally informative foraging strategies.” This reference to a “mental map” in *C. elegans* is striking: does this model predict that as a result of this search strategy, the neuronal network acquires an internal representation of the environment?

6) “The temporal dynamics of this probability, and the fact that it represents the accumulating evidence, suggests that a drift-diffusion model may be able to approximate this log-probability.” An immediate jump to a drift-diffusion model might not occur to me. What are the arguments for using this model (other than that it seems to work post factum, e.g., reproduce the linear slope, etc.)? This model serves as a basis of approximation, so what exactly is being “approximated” in this approach, i.e., what is disregarded and what is kept? The authors say “Ultimately, maximally informative trajectories could potentially exhibit a much greater degree of diversity than can be accounted for by the simpler drift-diffusion model.” So what do we sacrifice to get the approximation?

7) “Invariance properties that characterize the maximally informative foraging trajectories” – what is meant by “invariance” here?

8) “Although initial descriptions of the model suggested that the reduction in entropy associated with each discrete number of odorant hits should be computed, in our experience any approximation of this results in unwanted behavior. Therefore, we limited the maximal number of odorants that can be simultaneously detected to 1”. This paragraph is hard to parse, please explain more clearly.

9) Am I correct in understanding that optimal infotaxis results are shown in both Figures 5a and 5b?

10) I assume the red and black curves in Figure 6b follow the same convention as Figure 5a (but not of later panels in this figure) but it would be helpful to explicitly state what the color convention is in the caption for Figure 6b.

# Reply: Author response

**DOI:** 10.7554/eLife.04220.012

*1) However, there are some few issues that the reviewers did not fully understand, and I agree: is the drift-diffusion model described by* *Equation (3)* *connected to the worm's sensory data in some way*?

Authors: In the manuscript, we fit the drift-diffusion models to parameters of the infotaxis trajectories. Because these parameters also match experimental data, they could also be thought as a fit to experimental trajectories. We have added a discussion of this point.

The more general implication is that the sensory data sets the parameters of the drift-diffusion model. First, a sensory signal of odorant withdrawal starts the timer for the evidence accumulation in the drift-diffusion model. The drift rate depends on the parameters of food distribution that animals are acclimated to (e.g. small or wide patch of food). The dependence of the drift rate on the width of food patches matches what is needed to maximize mutual information. This drift rate is likely to be encoded using neuromodulatory signals (likely dopamine) that would integrate sensory data over much longer time scales to determine the environmental context for foraging conditions.

*In particular, decisions are reached when x reaches a value of zero, but is this initial value of x at the start of the local search? One of the reviewers thinks this is not clearly stated, and that might not be true, and I agree*.

The starting value for variable *x,* which represents the probability that the food is located within the local search area, was initially set to be slightly below 1. If the probability is set to exactly 1, meaning that the probability that the food source is outside the local search area, then it will never change. However, even when the deviations of this probability from 1 are as small as 10^-100^ the transition from the local to global search takes place (as in Figure 2C where log(1-x) is plotted). We have now clarified this point in the manuscript.

*2) One of the reviewers thinks that the qualitative features (e.g., the abrupt change from local to global search; food concentration independence of optimal infotaxic search) show that this model is distinct from chemotaxis, and that it would be helpful to make direct comparisons with best fits from situations of chemotaxis in the figures (**Figures 4a,b, 5B and 6b**)*.

We now provide the corresponding comparisons between measurements and best predictions of the chemotaxis model in Figures 4, 5b, 6b. The chemotaxis model makes qualitatively different (and in some cases opposite) predictions compared to the infotaxis model. For example, in Figure 5b, infotaxis predicts no change in animals’ behavior following the dilution of bacteria concentration, whereas the chemotaxis model predicts a decrease in the number of turns. In Figure 6b that compares the distribution of local search durations as a function of the width of the food patch, the infotaxis model predicts longer durations for wider patches whereas the chemotaxis model predicts shorter durations. The infotaxis predictions match experiments. Because Figure 4 appears in the manuscript much before we discuss the chemotaxis model, we first show this figure without the chemotaxis predictions, and then show the same data with chemotaxis predictions as Figure 4–figure supplement 1.

*Minor comments/questions*:

*1) How similar are the foraging behaviors in other invertebrate species mentioned, and are there any other instances of infotaxis/mutual information searching in animal behaviors other than visual saccades and bacteria*?

We have added references on foraging behaviors of ants, crabs, and bees that demonstrate qualitatively similar features of foraging that we describe here for *C. elegans*. For example, when ants that have been displaced on their way to the nest search the presumed nest location for a while but then transition to what could be equivalent to the global search. We have expanded this discussion point in the manuscript. There are only a handful published examples of trajectories after sufficiently large displacements that ants do not find their nest during the local search phase. We also hope to study foraging behaviors of the zebrafish larvae soon, which would provide an example of foraging in three dimensions.

*2) It would be interesting if the authors could describe an intuitively appealing rationale for why infotaxis as a method should be predicted to work. For example, when considering foraging behavior and various possible mathematical descriptions, is there a good a priori reason to expect that mutual information search would be favored*?

We now include the motivation for the use of mutual information in the Introduction section. To briefly restate the argument here, a number of measures other than the mutual information can be used to quantify a decrease in the uncertainty of the target position during foraging. These include the variance of its probability distribution or the height of the peak of this distribution. However, mutual information is unique in that it does not assume a certain set up of the foraging problem or properties of the distribution describing the distribution of food in the environment. For example, variance as a measure is only technically valid for Gaussian probability distributions of food locations. Once the probability distribution becomes non-Gaussian (this happens often after the exploration near the initial peak of the distribution), using variance to characterize the distribution leads to misleading results. Thus, the mutual information provides a more general measure to quantify changes in the probability distribution under a variety of circumstances, although one can use other measures in specific cases.

*3) The model contains three independent parameters that are fitted to reproduce the experimental distribution of the animal's positions at the end of the local search. Then the authors write, “Importantly, the same set of parameters can also account for the cumulative distribution of the durations of local search (**Figure 4b**).” Do they mean that the same set of parameters can be used to do this or that the same numerical values of the parameters account for the optimal durations*?

This sentence has been re-written to clarify that the same numerical values accounted for both probability distributions in Figure 4a and 4b.

“Importantly, the same set values of these parameters adjusted to match the spatial distribution (Figure 4a) also produced (without re-adjustment) cumulative distribution of local search duration that matched experimental measurements (Figure 4b, two-sample Kolmogorov-Smirnov test, *p*=0.45).”

*4) “Predictions of the infotaxis model are based on the relative distribution of food across space, rather than on its absolute concentration.” Sounds a bit confusing; chemotaxis is based on a gradient, not an absolute concentration*.

The sentence and paragraph that contained it has now been re-written to address this question as follows:

“The chemotaxis model makes predictions based on the change in odorant concentration. This change will be smaller for animals that are removed from patches with more diluted food. Therefore, the chemotaxis model in this case would predict that animals will make smaller number of turns (Figure 5b). In contrast, the infotaxis model makes predictions based not on the last odorant concentration value that the animal has experienced prior to its removal from food, but on the relative distribution of food in the environment. The spatial variance of this distribution is not affected by the dilution. Therefore, the infotaxis model would predict that the animals from regular and diluted bacteria lawns will make the same number of turns. This prediction was supported by our measurements (Figure 5b).”

*5) “At first glance, these calculations require the ability to maintain and update such “mental maps”, which may seem too computationally demanding to be implemented in a small neural circuit, such as the one found in* C. elegans*. Therefore, one may wonder whether the animals approximate the maximally informative foraging strategies.” This reference to a “mental map” in* C. elegans *is striking: does this model predict that as a result of this search strategy, the neuronal network acquires an internal representation of the environment*?

This was indeed our initial supposition. However, given that both the infotaxis and drift-diffusion models account for the data, at present one can only state that animals estimate the variance of food distribution in space and adjust search times accordingly. The fact that search times are adjusted in ways that maximizes mutual information supports the argument for information maximization as the behavioral goal. Future experiments with more detailed monitoring of animals’ movements could provide an indication that animals estimate high-order parameters of the food distribution. We have added this discussion point in the manuscript.

*6) “The temporal dynamics of this probability, and the fact that it represents the accumulating evidence, suggests that a drift-diffusion model may be able to approximate this log-probability.” An immediate jump to a drift-diffusion model might not occur to me. What are the arguments for using this model (other than that it seems to work post factum, e.g., reproduce the linear slope, etc.)? This model serves as a basis of approximation, so what exactly is being “approximated” in this approach, i.e., what is disregarded and what is kept? The authors say “Ultimately, maximally informative trajectories could potentially exhibit a much greater degree of diversity than can be accounted for by the simpler drift-diffusion model.” So what do we sacrifice to get the approximation*?

Our main goal was to account for the timing of the transition between local and global search. We tried many different criteria, including the entropy of the likelihood distribution of food source location at the decision time, local variance of this distribution near the decision point, direction of the gradient, etc. However, none of these measures could reliably predict the switching point between local and global phases of the search, until we looked at the normalization constant representing that the likelihood of finding food elsewhere. When this probability reached 1, the switch from local to global phase of the search occurred. The drift-diffusion model captures both the approximately linear increase and stochastic properties of foraging.

The simplified drift-diffusion model has only one parameter; the drift rate. Thus, for a given odorant, the drif-diffusion model can only encode one parameter of typical food patches, i.e. their size. To modify their behavior according to additional high-order parameters of the food patch distribution, the animals will have to use more complex models. We have expanded the discussion of these two points in the manuscript.

*7) “Invariance properties that characterize the maximally informative foraging trajectories” – what is meant by ”invariance” here*?

We have now clarified at this point in the text that invariance refers to the independence of the search strategies to the concentration of food within the patch. We also no longer use the term “invariance” to avoid confusion.

*8) “Although initial descriptions of the model suggested that the reduction in entropy associated with each discrete number of odorant hits should be computed, in our experience any approximation of this results in unwanted behavior. Therefore, we limited the maximal number of odorants that can be simultaneously detected to 1”. This paragraph is hard to parse, please explain more clearly*.

We have re-written this paragraph to clarify that we use binary representation separating odorant detection events into zero hit event and non-zero hit events. Keeping track of separate probability of observing 1,2,3, etc. hits at a time led to numerically unstable results.

*9) Am I correct in understanding that optimal infotaxis results are shown in both* *Figures 5a and 5b*?

We have added predictions based on infotaxis and chemotaxis models to Figure 5b. Panel A shows that measured trajectories (red line) are not consistent with different possible chemotaxis models.

*10) I assume the red and black curves in* *Figure 6b* *follow the same convention as* *Figure 5a* *(but not of later panels in this figure) but it would be helpful to explicitly state what the color convention is in the caption for* *Figure 6b*.

That is correct. We have now clarified that the panels A and B of Figure 6 follow the same color convention that is different from the color convention used in panels C-F.
